# Supplementary material for: A homozygous human WNT11 variant is associated with laterality, heart and renal defects
Source: Dis Model Mech. 2025 May 14;18(5):dmm052211. doi: 10.1242/dmm.052211 (PMC12091873; doi:10.1242/dmm.052211)
Supplement: Supplementary information [file dmm-18-052211-s1.pdf]

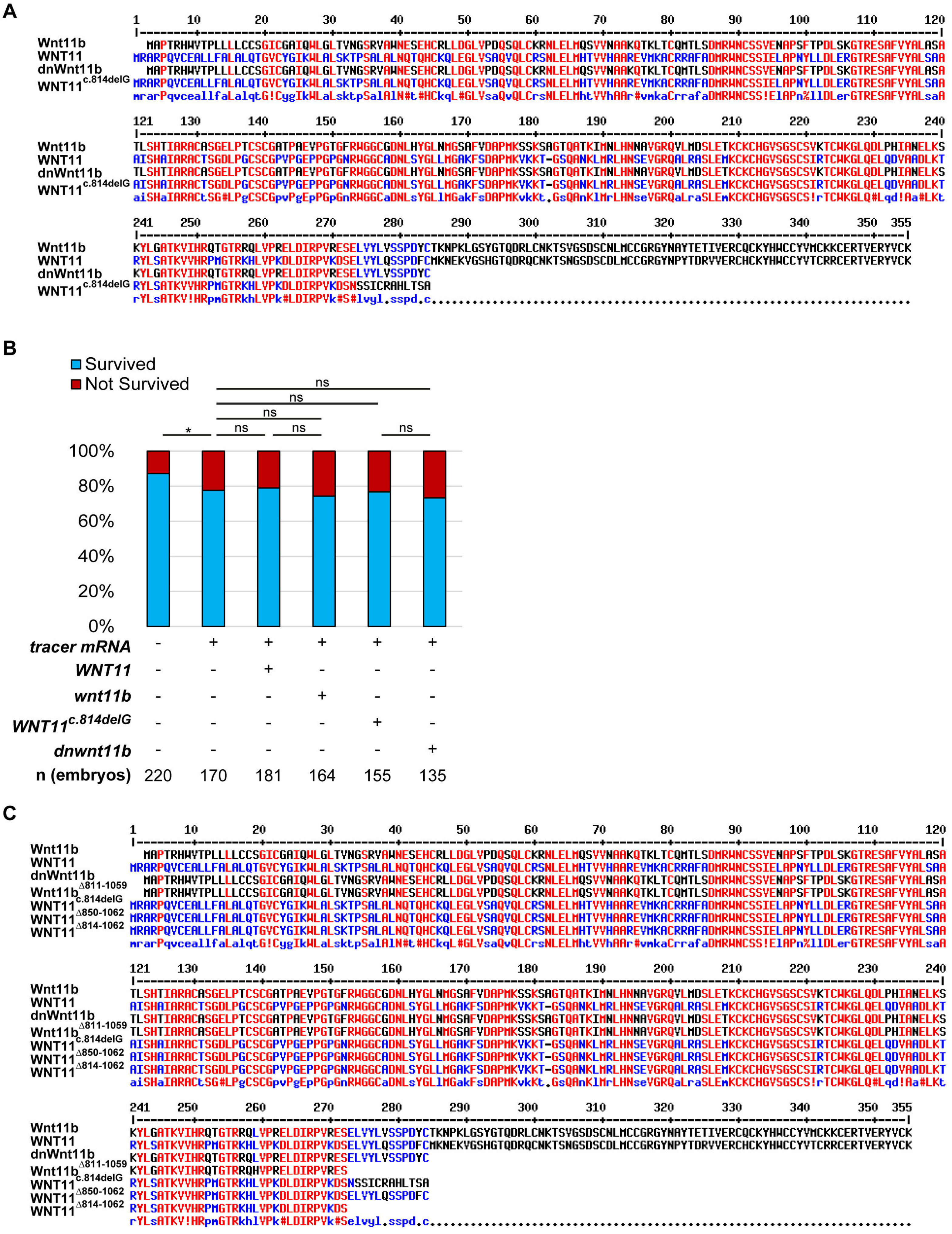

Fig. S1. Sequence comparison and mortality rates. (A) Alignment of nucleotide sequences of *WNT11, WNT11$^{c.814delG}$, Wnt11b and dnWnt11b*. **(B)** Mortality rates recorded in experiments corresponding to main figure 1A,B. Embryos were derived from 6 different batches (different parents) for Uninj. Ctrl., Inj. Ctrl., WNT11, WNT11$^{c.814delG}$, and from 5 different batches for wnt11b and dnwnt11b conditions. **(C)** Alignment of amino acid sequences of WNT11, WNT11$^{c.814delG}$, Wnt11b and dnWnt11b.

Disease Models & Mechanisms • Supplementary information

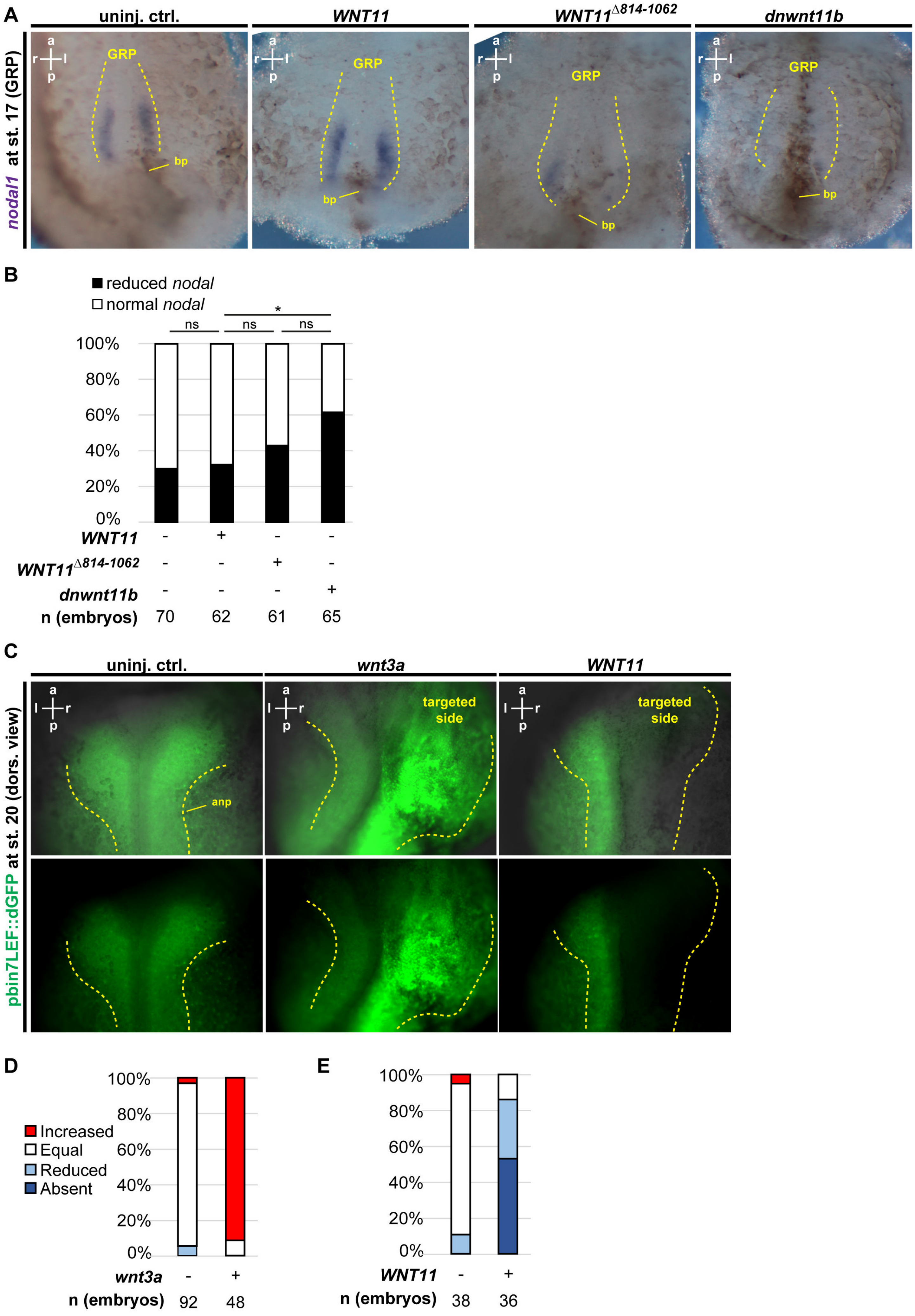

Disease Models & Mechanisms • Supplementary information

Fig. S2. Effects on *nodal* expression and validation of Wnt-reporter sensitivity. (A-B) Overexpression of DNAs (at 2ng/ml) encoding WNT11, WNT11$^{D814-1062}$ and dnWnt11b, and analysis of effects on *nodal1* by WMISH at st. 17/18. **(A)** Representative images. In **(A)**, GRPs are outlined by yellow dashed lines; blastopores (bp) are indicated. Ventral views on dorsal-posterior archenteron. **(B)** Quantification of results. n = number of embryos analyzed per condition. C^2-test, $p > 0.05$ = ns; $p < 0.05$ = *. In **(B)**, embryos were derived from 3 different batches (different parents) for all conditions. **(C-E)** Unilateral, right-sided overexpression of *wnt3a* mRNA (at 5ng/ml) or *WNT11* (100ng/ml) and analysis of effects on Wnt/b-catenin signaling reporter (pbinLEF::dGFP) activity (green). **(C)** Representative images. **(D)** Quantification of Wnt3a results. n = number of embryos analyzed per condition. **(E)** Quantification of WNT11 results. n = number of embryos analyzed per condition. In **(C)**, embryos were derived from 3 different batches (different parents). In **(D,E)**, embryos were derived from 2 different batches (different parents).

Disease Models & Mechanisms • Supplementary information

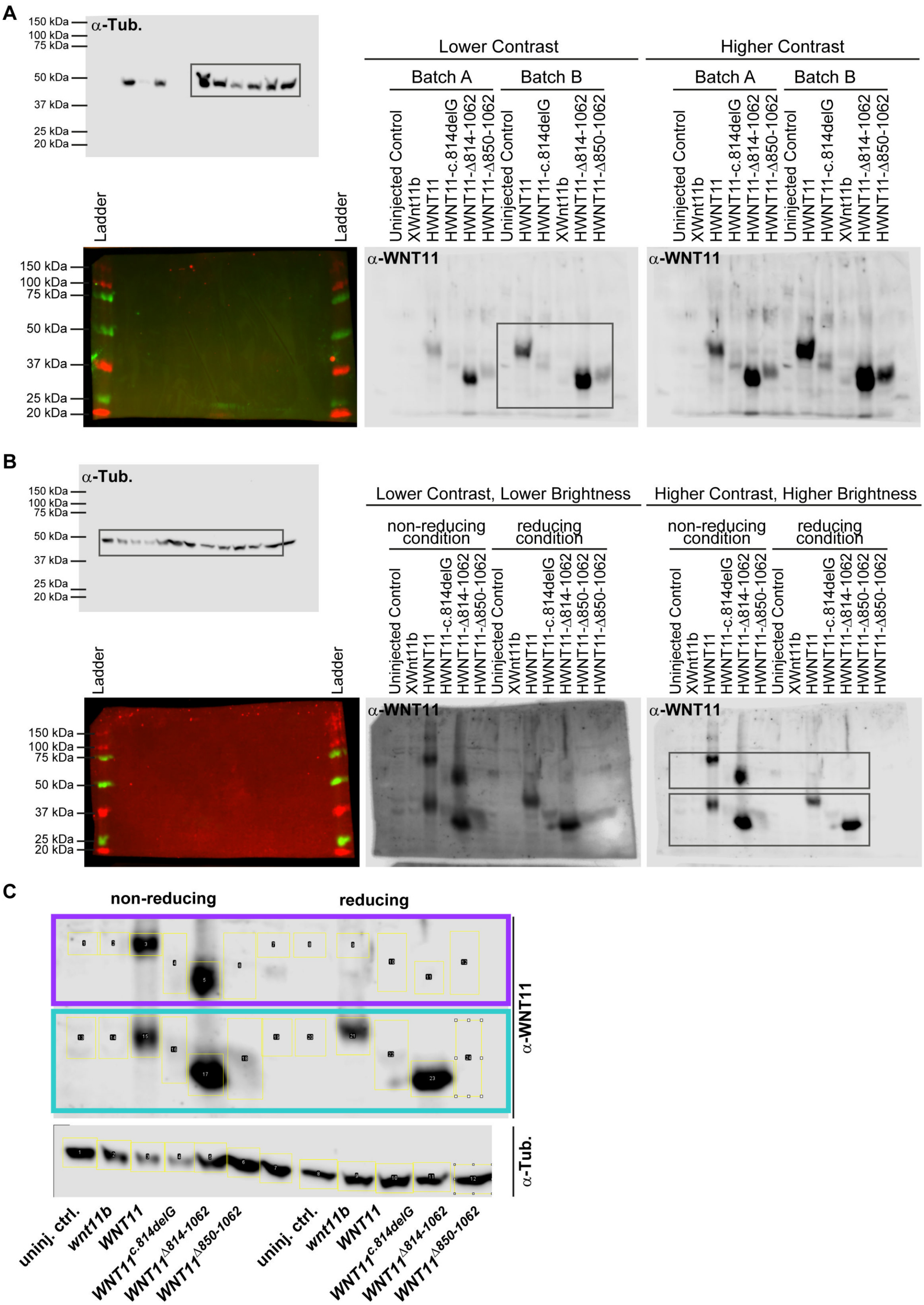

Fig. S3. Western blot membranes and quantification areas. (A) Full membranes of experiments corresponding to main figure 4C. **(B)** Full membranes of experiments corresponding to main Fig. 4D. **(C)** Areas (ROIs) used for quantification of protein levels corresponding to graphs in main figure 4D. In **(A)** and **(B)**, the membrane areas shown in main Fig. 4C, D are outlined by black boxes.

Disease Models & Mechanisms • Supplementary information

Table S1. Primers used for Sanger sequencing (5′-3′).

Name	Sequence
WNT11_F1	5′-GCCTAATTCGAGCCAGGCG-3′
WNT11_R1	5′-CGGGAGGCAGGGACATTTTA-3′
WNT11_F2A	5′-GCTGACAGTTTGGGAGCCTT-3′
WNT11_R2A	5′-AGCAAATAGTTGGGGGCGAG-3′
WNT11_F2B	5′-GGAGGGTCTGGTGTCTGCAC-3′
WNT11_R2B	5′-ATGCCTGCACACCCATATTTATGAT-3′
WNT11_F3	5′-ACCTTGGTTACAGCAGGGTG-3′
WNT11_R3	5′-TTGGAGGAGGAAAGCGACAC-3′
WNT11_F4	5′-CCACACCACCTCCAAGCTTTA-3′
WNT11_R4	5′-CTGAGCAGGGTCTCCATTCC-3′
WNT11_F5	5′-TAAGAAGGGCTGAGTCGGTG-3′
WNT11_R5	5′-TTCTGTTCCTGGTGGCTTCC-3′

Table S2. Cloning primers to generate new WNT11 and Wnt11b constructs (5′- 3′).

Name	Sequence
Cla1-WNT11-F	5′-AAAAAAATCGATATGAGGGCGCGGCCCAGGT-3′
WNT11-EcoR1-R	5′-AAAAAAGAATTCTCACTTGCAGACATAGCGCT-3′
WNT11-c.814delG F	5′-AACTCGTCTATCTGCAGAGC-3′
WNT11-c.814delG R	5′-CGAGTCCTTCACAGGCCG-3′
WNT11-Δ814-1062 F	5′-TGAGAATTCGTCGACAGGC-3′
WNT11-Δ814-1062 R	5′-CGAGTCCTTCACAGGCCG-3′
WNT11-Δ850-1062 F	5′-TGAGAATTCGTCGACAGG-3′
WNT11-Δ850-1062 R	5′-GCAGAAGTCAGGTGAGCT-3′
wnt11b-Δ811-1059 F	5′-TAACTCGAGCCTCTAGAAC-3′
wnt11b-Δ811-1059 R	5′-AGACTCTCTCACTGGCCT-3′

Disease Models & Mechanisms • Supplementary information

Table S3. Areas and intensity values of ROIs of bands.

ROI #	WNT11 area	WNT11 intensity	ROI #	α-Tub area	α-Tub intensity
1	0.012	34.279	1	0.016	109.311
2	0.012	38.218	2	0.016	83.963
3	0.012	180.884	3	0.016	57.081
4	0.023	36.698	4	0.014	54.226
5	0.019	187.286	5	0.015	108.097
6	0.035	32.213	6	0.015	135.56
7	0.013	30.066	7	0.018	116.032
8	0.013	30.002	8	0.018	82.809
9	0.013	36.180	9	0.018	90.851
10	0.027	30.003	10	0.018	104.415
11	0.015	30.965	11	0.018	87.628
12	0.030	30.000	12	0.018	106.712
13	0.019	36.478			
14	0.019	32.600			
15	0.019	134.917			
16	0.027	40.680			
17	0.024	191.360			
18	0.048	49.123			
19	0.019	30.012			
20	0.019	30.000			
21	0.020	108.885			
22	0.038	37.789			
23	0.025	161.967			
24	0.032	30.619			

Disease Models & Mechanisms • Supplementary information
